# The impact of having a dedicated obstetrics and gynecology resident to provide contraceptive counseling on immediate postpartum family planning uptake: a “pre–post” study

**DOI:** 10.1186/s12978-022-01361-6

**Published:** 2022-03-03

**Authors:** Abraham Fessehaye Sium, Mekdes Wolderufael, Don Eliseo Lucero-Prisno, Jaclyn M. Grentzer

**Affiliations:** 1 Department of Obstetrics and Gynecology, St. Paul’s Hospital Millennium Medical College (SPHMMC), Addis Ababa, Ethiopia; 2 Department of Global Health and Development, London School of Hygiene and Tropical Medicine, London, UK; 3 Faculty of Management and Development Studies, University of the Philippines (Open University), Los Baños, Laguna Philippines

**Keywords:** Immediate postpartum family planning, Postpartum family planning uptake, Family planning counselling, Modern contraception

## Abstract

**Background:**

Providing effective, high quality, antenatal and postpartum contraceptive counseling can reduce unintended pregnancies, decrease maternal and fetal morbidity and mortality, and prevent unsafe abortions. The postpartum period is a critical time to address unmet family planning need and to reduce the risks of short interpregnancy interval. This study aimed at determining the impact of assigning a dedicated obstetrics and gynecology resident for postpartum family planning counselling on the uptake of immediate postpartum family planning.

**Methods:**

A “pre-post” observational study was conducted at Saint Paul’s Hospital Millennium Medical College (SPHMMC), in Addis Ababa-Ethiopia, from May 1, 2021 to June 30, 2021. Immediate postpartum family planning uptake between the months of June (when there was a dedicated resident assigned for postpartum family planning counselling and provison on weekdays) and May (when there was no such dedicated resident for similar purpose) were compared. Data was analyzed using SPSS version 20 software packages. Simple descriptive was used to describe baseline characteristics. Chi-square test of association was done to determine the correlation between dependent and independent variables. Multivariate regression analysis was applied to determine factors associated with uptake of family planning methods in the immediate postpartum period. Odds ratio, 95% CI, and p-value < 0.05 were used to describe results significance.

**Results:**

Out of 776 mothers who delivered at SPHMMC in the month of June 2021, 158 (20.4%) of them used immediate postpartum family planning. This finding during the month of June is higher than a 15.4% immediate postpartum family planning uptake observed during the preceding month of May. Having a dedicated resident for postpartum family planning counselling was associated with an increase in immediate postpartum family planning use (AOR = 1.31, 95% CI [1.01, 1.69]).

**Conclusion:**

In this study, presence of a dedicated obstetrics and gynecology resident for postpartum family planning counselling was associated with an increase in the uptake of immediate postpartum family planning. This implies the importance of assigning a dedicated care provider for the purpose of postpartum family planning counselling within the immediate postpartum, which gives postpartum women another opportunity of adequate counselling before they are discharge from Hospitals or obstetric service centers.

## Plain Language summary

The postpartum period is a critical time to address unmet family planning need and to reduce the risks of short interpregnancy interval and unintended pregnancies, which translates in to a decrease in maternal and fetal morbidity and mortality. In this study, postpartum women who were counselled for family planning by dedicated obstetrics and gynecology resident were more likely to use immediate postpartum family planning (PPFP) compared to women who were not counselled by such care provider. This study aimed at determining the impact of assigning a dedicated obstetrics and gynecology resident for postpartum family planning counselling, by comparing immediate PPFP (family planning provided after delivery and before discharge of mothers from Hospital) uptake between the months of June (when there was a dedicated resident) and May (when there was no dedicated resident), in 2021. Out of 908 deliveries during the month of May, the uptake of immediate PPFP was 15.4%, which was significantly lower than an uptake of 20.4% in the following month of June. Mothers who delivered during the month of June were 1.3 times more likely to use immediate PPFP than mothers who delivered in the preceding month of May. In conclusion, presence of a dedicated obstetrics and gynecology resident for postpartum family planning counselling was associated with an increase in the uptake of immediate postpartum family planning.

## Introduction

Giving mothers the opportunity to time their pregnancies can reduce maternal and child mortality by 30% and 10%, respectively [1, 2]. In addition, contraceptive use within the first 12 months after childbirth has the potential of reducing the risk of undesired pregnancy outcomes, such as preterm birth, low birth weight and small size for gestational age [3–5]. Due to the low prevalence of postpartum contraception [6], women in Africa are still victims of these undesired pregnancy outcomes and high maternal mortality rate [7, 8].

The postpartum period is a critical time to address unmet family planning need and to reduce the risks of short interpregnancy interval [8–10]. Providing effective, high quality, antenatal and postpartum contraceptive counseling can reduce unintended pregnancies, decrease maternal and fetal morbidity and mortality and prevent unsafe abortions [11]. A recent systematic review and meta-analysis found that postpartum family planning uptake in Ethiopia is low compared to the existing global recommendations on postpartum contraceptive use. Resumption of sexual activity, having received antenatal care, maternal education above or equal to secondary school, postnatal care with family planning counselling, resumption of menses, and being ≥ 6 months postpartum were found to be significantly associated with postpartum contraceptive use [12].

Family planning has been included as part of the curriculum for post-graduate obstetrics and gynecology residency training at St. Paul’s Hospital Millennium Medical College in Addis Ababa Ethiopia, where at residents have a monthly family planning attachment during their stay in the residency training. This study aimed at determining the impact of having a dedicated obstetrics and gynecology resident for postpartum family planning counselling on uptake of immediate postpartum family planning.

## Methods

This is a “pre–post” observational study conducted at Saint Paul’s Hospital Millennium Medical College (SPHMMC) in Addis Ababa, Ethiopia, from May 1–June 30 2021. SPHMMC is a teaching hospital and one of the tertiary national referral hospitals in Ethiopia. It attends one of the highest deliveries in Ethiopia, monthly there are 800–900 deliveries at the Hospital. It is also a center of excellence for family planning (FP) service provision and FP is part of the curriculum for both undergraduate and post-graduate students at the college. Postpartum family planning (PPFP) counselling is provided to women who deliver at the Hospital by primary care providers who attend their deliveries. Moreover, during their monthly attachment as part of their residency training, obstetrics and gynecology residents are assigned to family planning unit  to improve the adequacy of postpartum family planning counselling, through giving all postpartum women a second opportunity of counselling—capture those who were not counselled and counsel again those who are already advised for FP but didn’t take any method. Infrequently,  this assignment is interrupted due to shortage of residents, as it occurred during the month of May 2021. The only intervention introduced during  the month of Junewas restarting the practice of assigning a dedicated resident for PPFP counselling.

In this study, we aimed at determining the impact of having  a dedicated obstetrics and gynecology resident for postpartum family planning counselling on postpartum family planning uptake.  We compared the uptake of immediate postpartum family planning uptake between the months of June (when there was a dedicated resident) and May (when there was no dedicated resident) 2021. Immediate postpartum family planning is defined in this study as modern contraception provided after delivery and before discharge of mothers from Hospital, while dedicated obstetrics and gynecology resident is described as a resident assigned for the solo purpose of postpartum family planning counselling on weekdays (Monday–Friday, excluding weekend and Holidays, from 8:00 AM–4:30 PM). All women who delivered at SPHMMC during the months of May and June 2021 were included in the study. Data on immediate postpartum family planning uptake, family planning methods, mode of delivery, delivery to family planning uptake interval, date of delivery, and socio-demographic characteristics was extracted from labor and delivery wards’ birth and family planning registries, and post-natal wards’ family planning registry, using a data extraction form prepared in English language. Immediate postpartum family planning uptake and types of modern contraception used were the dependent variables while independent variables comprised of presence of dedicated resident, maternal age, mode of delivery, address, day of delivery (working day vs holidays and weekend), delivery to family planning use interval in days, and type of client (new client vs returning client).

Data was collected by two obstetrics and gynecology residents, entered into Epi-info version 3.5.1 and later exported to SPSS version 20 software packages for analysis. Simple descriptive was used to describe baseline characteristics. Chi-square test of association was carried out  to determine the correlation between dependent and independent variables. Multivariate regression analysis was applied to determine factors associated with uptake of immediate postpartum family planning (PPFP). Odds ratio, 95% CI, and p-value < 0.05 were used to present results significance.

## Results

In this study, out of 1684 deliveries (908 deliveries recorded in May 2021 and 776 deliveries recorded in June 2021), the uptake of immediate PPFP was 17.7% (Table 1). Around twenty percent of mothers who delivered in the month of June used family planning in the immediate postpartum (Table 1). This is significantly higher than immediate PPFP coverage of 15.4% observed during the preceding month of May, p-value = 0.008. This finding translates in to an increase in the uptake of Immediate PPFP by 12.9%, which is statistically significant (Table 2). And when the immediate PPFP uptake during weekdays between the 2 months were compared, there was an increase by 25.2% during the month of June, which is statistically significant (p-value = 0.000).

**Table 1:** Baseline characteristics of all mothers who delivered during the months of May and June (2021)

Characteristics	May No dedicated resident		June + Dedicated resident		Increment	Total	
	n	%	n	%	%	n	%
**Immediate PPFP Status **							
Didn’t use	768	84.6	618	79.6	− 19.5	1386	82.3
Used	140	15.4	158	20.4	12.9	298	17.7
**Date of PPFP uptake**							
Weekday	123	13.5	154	19.9	25.2	277	16.5
Weekend and Holiday	17	1.9	3	0.4	− 82.4	20	1.2
Didn’t use	768	84.6	618	79.7	− 19.5	1386	82.4
**Type of PPFP client**							
New client	88	9.7	101	13.0	14.8	189	11.2
Returning client	52	5.7	56	7.2	7.7	108	6.4
**Type of family planning method used**							
Implanon NXT	121	13.3	126	16.2	4.1	247	14.7
Jadelle	18	2.0	24	3.1	33.3	42	2.5
IUD	1	0.1	5	0.6	400	6	0.4
BTL	0	0	3	0.4	–	3	0.2
**Mode of delivery**							
Vaginal	483	53.3	342	44.3	− 29.2	825	49.2
Instrument	36	4.0	20	2.6	− 44.4	56	3.3
CS	387	42.7	410	53.1	5.9	797	47.5
**Address**							
Addis Ababa	470	52.0	461	59.6	− 1.9	931	55.5
Outside of Addis Ababa	434	48.0	313	40.4	− 27.9	747	44.5
**Age**							
Mean	26.5 (17–42)		26.9 (17–43)			26.7

**Table 2:** Immediate postpartum family planning uptake in relation to time, FP method, and type of client among Family planning (FP) users

	May (No dedicated resident)		June (+ dedicated resident)		Increment	p-value
	n	%	n	%	%	
**Date of PPFP uptake**						
Weekday	123	87.9	154	98.1	25.2	0.000*
Weekend and holyday	17	12.1	3	1.9	− 82.4	
**Delivery to PPFP uptake interval in day**s						
1st postpartum day	117	83.6	107	67.7	− 8.5	0.002*
2nd and 3rd postpartum day	23	16.4	51	32.3	121.7	
**Type of PPFP client**						
New client	88	62.9	101	64.3	14.8	0.792
Returning client	52	37.1	56	35.7	7.7	
**Type of family planning method**						
Implanon NXT	121	86.4	126	79.7	82.9	0.135
Jadelle	18	12.9	24	15.2	14.1	
IUD	1	0.7	5	3.2	2.0	
BTL	0^1^	0.0	3	1.9	1.0	
**Mode of delivery**						
Vaginal	59	42.4	40	25.3	− 32.2	0.007*
CS	78	56.1	116	73.4	48.7	
Instrument	2	1.4	2	1.3	0	
**Address**						
Addis Ababa	64	46.0	96	61.5	50.0	0.008*
Outside of Addis Ababa	75	54.0	60	38.5	− 20.0	
**Age**						
Mean	26.4 (17–39)		27.5 (20–38)			

When it comes to the family planning methods used (Table 2), Implanon NXT was the most commonly accepted method during both months, representing 86.4% and 79.7% of the total immediate postpartum family planning provided during the months of  May and June respectively, which was not statistically significant. Analysis of family planning uptake according to the delivery to family planning uptake time interval showed a sharp increase (by 121.7%) in the uptake of immediate PPFP in the 2^nd^ and 3^rd^ postpartum days. It jumped from 16.4% in May to 32.3% in June (p-value 0.002). Women who delivered through cesarean section (CS) represented majority of the immediate PPFP users during both months (73% in the month of June and 56.1% in the month of May, respectively), which is also statistically significant (p-value = 0.007).

Multivariate regression analysis revealed that mode of delivery and having a dedicated resident for postpartum family planning counselling were significantly associated with increased immediate PPFP uptake (Table 3). Mothers who delivered during the month of June were 1.3 times more likely to take immediate postpartum family planning than mothers who delivered in the month of May (AOR = 1.31, 95% CI [1.01, 1.69]). Mothers who delivered vaginally were 56.2% (AOR = 0.438, 95% CI [0.34, 0.57]) less likely to use immediate postpartum family planning than mothers who deliver by CS. Likewise, mothers who had instrumental deliveries were 74.5% less likely to use such family planning than mothers who delivered by CS (AOR = 0.255, 95% CI [0.09, 0.72]).

**Table 3:** Multivariate logistic regression analysis of factors associated with immediate PPFP uptake

Variable	AOR	95% CI
**Age**	1.009	0.981–1.038
**Mode of delivery**		
Vaginal	0.438	0.335–0.573
Instrument	0.255	0.091–0.715
CS	1	
**Delivery date**		
May (No dedicated resident)	1	
June (+ Dedicated resident)	1.305	1.009–1.689
**Address**		
Addis Ababa	0.885	0.682–1.148
Outside of Addis Ababa	1	

## Discussion

Contraceptive use has reduced maternal death by 40% worldwide in the last 25 years. An additional 30% decrease in maternal deaths in developing countries could be achieved if all women who wanted to avoid pregnancy used an effective contraceptive method [2]. Use of LARC methods in the immediate postpartum period is both effective and safe, and could reduce unmet need for contraception during this time [13]. Despite this evidence, up to 61% of women in low-income and middle income countries are not using effective contraception within 24 months postpartum to avoid an unintended pregnancy [14]. Postpartum family planning (PPFP) uptake remains low in East Africa [15]. The estimated unmet need for contraception for married women in sub-Saharan Africa is 27.7% [16–18]. Between 2011 and 2016, the postpartum contraceptive prevalence in Ethiopia increased from 15 to 23% [19]. A recent study done in Ethiopia (Addis Ababa) found that 45% of postpartum women accepted long-term and permanent contraception on their immediate postpartum period before discharge [20]. In the present study, postpartum family planning uptake in the immediate postpartum(from the time of delivery till discharge from Hospital) over 2 months period was 17.7%, which is comparable to 19% reported in a study done in Northwest of Ethiopia [21] but much lower than 63% found in a recent study done in Northwest Ethiopia [22], 44% in south Ethiopia [23], 46% in studies done in Kenya and Zambia [24], and 50% reported from Rwanda [25].

According to a recent systematic review of 19 studies (11 out of which were from Ethiopia), unmet needs in postpartum FP in women from Sub-Saharan Africa were associated with health-system and socio-demographic determinants. It is also suggested that there is a need to improve the awareness of modern contraceptive methods through effective interventions [26]. In this study, a significant improvement in the uptake of immediate PPFP was observed after assigning a dedicated obstetrics and gynecology resident for counselling. It climbed from 15.5% during the month of May to 20.4% in June (an increment by 12.9%). Further analysis of immediate PPFP uptake by date revealed that 98% of the uptake in the month of June occurred during weekdays as in contrast  to 87.9% during the month of May on the same days (a 25% increment). This association between having a dedicated resident and increased immediate postpartum family planning uptake remained unchanged after multivariate regression analysis was carried out . Mothers who gave birth during the month of June were 1.3 times more likely to use immediate PPFP than mothers who delivered in the month of May (AOR = 1.31, 95% CI [1.01, 1.69]). This finding in our study mirrors a finding from a previous study done in Ethiopia which showed that postpartum family planning counselling improves immediate postpartum family planning acceptance (OR = 2.13, 95% CI 1.004–3.331) [20].

The other significant finding in the present study is the association between the uptake and delivery to time of acceptance of postpartum family interval. The uptake of PPFP in the 2nd and 3rd postpartum days doubled in the month of June as compared to that of during the month of May (16.4% in May vs 32.3% in June, p-value = 0.002). This highlights the importance of repeated counselling in the second and thirddays postpartum, which was likely carried out by the assigned dedicated resident.

Moreover, in this study, mode of delivery was found to have an association with immediate PPFP uptake. Mothers who delivered vaginally were 56.2% (AOR = 0.438, 95% CI [0.34, 0.57]) less likely to take immediate PPFP than mothers who delivered through CS. Likewise, mothers who deliver via instrument delivery were 74.5% less likely to use immediate PPFP than mothers who delivered by CS (AOR = 0.255, 95% CI [0.09, 0.72]). These associations may be explained by the fact that post-cesarean women in our hospital have longer hospital stay (minimum of 3 days) than women who deliver vaginally, which could have given them more opportunity to be counselled for the second time at post-natal wards. In contrast, women who have vaginal deliveries at our hospital are discharged after 6 hours stay postpartum (unless they have any obstetric or medical complication) which could have led them to miss such an opportunity of being counselled on the subsequent postpartum days by the dedicated resident. An important limitation of this study is not being able to determine the rate of FP uptake in relation to frequency of counselling (one time counselling vs repeated counselling) and quality of counselling. Moreover, parity, antenatal care FP counselling, women’s level of awareness about contraception, women’s educational level and partner involvement in counselling were not controlled in the multimodal regression analysis. These factors are well known contributors to uptake of family planning and it’s missing in our study may have made our study more prone to bias. The other limitation of this study is shorter observation time—over 2 consecutive months. A longer observation time could have given this study the opportunity to get a larger study sample size and stronger results.

## Conclusion

In this study, presence of a dedicated obstetrics and gynecology resident for postpartum family planning counselling was associated with an increase in immediate PPFP uptake. This implies the importance of assigning a dedicated care provider for the purpose of postpartum family planning counselling in the immediate postpartum (in addition to counselling that may be given by primary care providers who attend deliveries), which gives postpartum women another opportunity of receiving adequate  counselling. We recommend a further analytic study to examine the consistence of this impact after controlling the known contributors to postpartum family planning uptake, which were not analyzed in our study.

## Data Availability

All data generated or analyzed during this study are included in this published article.

## Abbreviations

AOR: Absolute odds ratio
CS: Cesarean section
CI: Confidence interval
IRB: Institutional review board
LARC: Long-acting reversible contraception
AOR: Absolute odds ratio
OR: Odds ratio
PPFP: Postpartum family planning
SPHMMC: St. Paul’s Hospital Millennium Medical College
